# Delivering Integrated Care to the Frail Elderly: The Impact on Professionals’ Objective Burden and Job Satisfaction

**DOI:** 10.5334/ijic.2014

**Published:** 2016-08-17

**Authors:** Benjamin Janse, Robbert Huijsman, Ruben Dennis Maurice de Kuyper, Isabelle Natalina Fabbricotti

**Affiliations:** 1 Rotterdam University of Applied Sciences, School of Health Care, P.O. Box 25035, 3001 HA Rotterdam, the Netherlands; 2 Institute of Health Policy and Management, Erasmus University Rotterdam, P.O. Box 1738, 3000 DR Rotterdam, the Netherlands; 3 Health Centre Arnemedi, Prins Bernhardstraat 2, 4341 EZ Arnemuiden, the Netherlands

**Keywords:** professionals, objective burden, job satisfaction, frail elderly

## Abstract

**Background:**

The impact of integrated working on professionals’ objective burden and job satisfaction was examined. An evidence-based intervention targeting frail elderly patients was implemented in the Walcheren region of the Netherlands in 2010. The intervention involved the primary care practice as a single entry point, and included proactive frailty screening, a comprehensive assessment of patient needs, case management, multidisciplinary teams, care plans and protocols, task delegation and task specialisation, a shared information system, a geriatric care network and integrated funding.

**Methods:**

A quasi-experimental design with a control group was used. Data regarding objective burden involved the professionals’ time investments over a 12-month period that were collected from patient medical records (n = 377) time registrations, transcripts of meetings and patient questionnaires. Data regarding job satisfaction were collected using questionnaires that were distributed to primary care and home-care professionals (n = 180) after the intervention’s implementation. Within- and between-groups comparisons and regression analyses were performed.

**Results:**

Non-patient related time was significantly higher in the experimental group than in the control group, whereas patient-related time did not differ. Job satisfaction remained unaffected by the intervention.

**Conclusion and Discussion:**

Integrated working is likely to increase objective burden as it requires professionals to perform additional activities that are largely unrelated to actual patient care. Implications for research and practice are discussed. [Current Controlled Trials ISRCTN05748494].

## Introduction

Integration is emerging as a central tenet of health systems [1]. As a result, professionals are increasingly required to deliver integrated care, particularly with respect to the growing population of community-dwelling frail elderly patients that are in need of complex and long-term care services from multiple organisations and providers [1,2,3]. Integrated care is generally defined as a ‘coherent set of methods and models on the funding, and the administrative, organisational, service delivery and clinical levels designed to create connectivity, alignment, and collaboration within and between the cure and care sectors’ [3]. For professionals, it involves a shift from the traditional and hierarchical organisation of care based on clinical disciplines towards patient-centred care delivery based on horizontal work processes [4,5,6]. To meet these new requirements, professionals must reshape their roles, practices and philosophies and need to be reshaped and professionals acquire new routines and methods [6,7,8,9,10]. It thus seems inevitable that integrated care places new demands on professionals and changes their daily work. The question arises whether these changes are to the benefit of professionals.

Integrated care delivery is widely believed to eliminate inefficiency and duplication in work processes and to relieve professionals of administrative tasks in favour of patient-related activities, reducing their time pressure –or ‘objective burden’ – and frustration [1,7,8,9,10,11,12]. Integrated working is assumed to contribute to a positive work environment by fostering inter-disciplinary collaboration and communication, and to increase job satisfaction by providing more opportunities for professional development and patient-centred care [10,11,12,13,14,15]. However, less favourable impacts may be equally plausible. Integrated care might hold considerable infringements upon the work, autonomy and identity of professionals, and its introduction might cause organisational upheaval, conflicts, deteriorating relationships and professional dissatisfaction [8,16,17,18]. Moreover, integrated working might actually hamper workflows and thus increase objective burden [9,10,11]. Common integration mechanisms, such as multidisciplinary meetings and a shared information system, are typically placed on top of existing structures rather than replacing them, resulting in duplication and inefficiency and making coordination among professionals increasingly time-consuming [10,11]. In addition, integrated working may require professionals to take on additional tasks alongside their day-to-day activities. For instance, the active recruitment of patients may increase patient flows, and collecting and documenting additional patient information involves actions that were not previously required [10,19]. As well, integrated working requires professionals to learn new tasks and to absorb them into existing routines, which is likely to demand additional inputs of time [1,7,16,17,18,19].

The perceptions and experiences of professionals in the integrated care context have been well documented over the years [6,7,9,11,12,13,16,17,18]. Reports on integrated working often describe professionals experiencing increased time demands, intensive workloads and productivity problems [20,21,22,23,24,25]. Similarly, studies suggest that integrated working causes a shift towards more non-patient related activities, such as administrative tasks and team meetings [26,27,28]. Whilst the concerns regarding integrated working thus seem justified, the current evidence is largely based on self-report and qualitative data. Understanding the objective impacts of integrated care on professionals requires detailed data from formal administrative systems [29]. Such data are, however, extremely sparse in the literature, as a result of which little is known regarding the objective burden of professionals delivering integrated care.

The present study aimed to fill this gap by performing a comprehensive analysis of professionals’ time investments during the first 12 months of integrated working. The research setting was the Walcheren region of the Netherlands, where an intervention was implemented that specifically targeted independently living frail elderly patients (and their informal caregivers). This ‘Walcheren Integrated Care Model’ was designed in accordance with the evidence at the time of implementation in 2010. In conjunction with the implementation of the intervention, a geriatric care network was created that included a hospital, a mental health organisation, allied health practices and patient, informal caregiver and volunteering associations. Representatives of the network partners, municipalities, and social care and welfare agencies formed a formal steering group was responsible for the planning and implementation of the intervention. The regional health insurer provided an experimental financial module to reimburse all intervention-related costs to participating primary care practices. The present study was part of a large-scale evaluation of the Walcheren Integrated Care Model. The following research question was used: What is the impact of the Walcheren Integrated Care Model on the objective burden and job satisfaction of professionals?

## Methods

### Study design and selection

The medical ethics committee of the Erasmus Medical Centre Rotterdam reviewed and approved the study protocol (No. MEC-2013-058). This study involved a quasi-experimental design with a control group. The experimental group consisted of 3 primary care practices located in eastern Walcheren that provided care according to the intervention, and the control group consisted of 5 primary care practices located in northern, southern and western Walcheren that provided care as usual. Control practices had not been involved in the intervention’s development and did not use any of the intervention components. The selection of control practices continued until the control and experimental group consisted of a similar number of frail elderly patients.

Inclusion criteria of patients were age (≥75) and frailty. Frailty was measured with the Groningen Frailty Indicator, a validated and widely used screening instrument [30]. Practices provided the names and contact information of the patients that met the inclusion criteria. Patients were subsequently mailed an information leaflet, the screening instrument, an informed consent form and a postage paid envelope. The researchers identified frail elderly patients based on their screening scores (≥64) and provided their names to the intervention practices. Control practices remained uninformed regarding the frailty of their elderly patients during the evaluation period. Exclusion criteria for patients were being on a waiting list for a nursing home and having a terminal illness with a life expectancy of less than 6 months.

### Intervention

**Figure 1:**
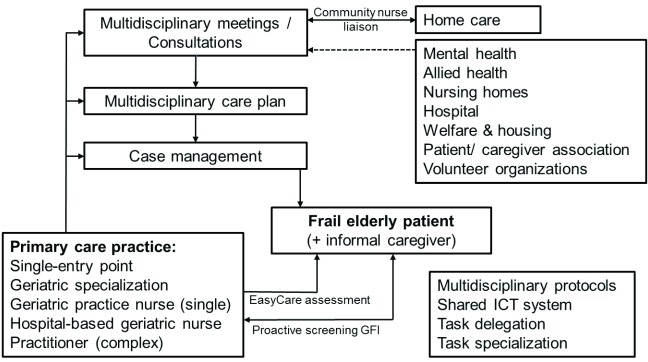
The Walcheren Integrated Care Model.

Care as usual in the Netherlands can be described as reactive. Patients usually consult with their general practitioner on their own initiative. Care and curative services can only be accessed through referral of their general practitioner [31]. Communication and information-exchanges between primary, secondary and tertiary professionals is typically bilateral and ad hoc. The aim of the Walcheren Integrated Care Model was to address these issues in the care for frail elderly patients in the community using the following components: a single entry-point, proactive screening, comprehensive needs assessments, case management, multidisciplinary group meetings, care plans and protocols, a shared information system, and tasks specialisation and tasks delegation (Figure 1). The primary care practice functioned as a *single entry point* for frail elderly patients, their informal caregivers and professionals. All elderly patients (75+) were *proactively screened*. Frail patients (frailty score ≥ 4; range 1–15) were visited by a case manager, who performed a *comprehensive assessment* of needs of patients and their informal caregiver(s) using an evidence-based assessment tool [32]. The results of the assessments were subsequently discussed in a *multidisciplinary group meeting* chaired by the general practitioner. The core team consisted of the general practitioner, a case manager and a community nurse employed by the home-care organisations. Home-care organisations provide various services in the patient’s home, ranging from around-the-clock supervision and/or specialized nursing care, home rehabilitation, home meal services, personal care and domestic assistance, using small community-based teams of general and specialized nurses and domestic helpers. The community nurse acted as a liaison for home-care professionals by relaying their wishes, observations and suggestions to the intervention team. This arrangement aimed to better utilize the unique information and signalling-function of home-care professionals, owing to their close proximity to patients and informal caregivers.

Other professionals relevant to the patient’s care trajectory, such as a hospital geriatrician, a nursing home doctor, a physiotherapist, a social worker or psychologist, could attend team meetings. The team formulated a *multidisciplinary care plan* in consultation with the patient and informal caregiver(s), after which tasks were assigned to the appropriate professionals according to *multidisciplinary care protocols. Case management* involved ensuring access to the appropriate services and planning, coordinating and monitoring care delivery. A specialised practice nurse performed ‘single-disease’ case management, whereas a hospital geriatric nurse-specialist performed ‘complex care’ case management. The care plan was periodically evaluated in a multi-disciplinary meeting, the frequency of which ranged from once a month to once a year, depending on the patient’s condition.

The entire process was supported by *task delegation*, task *specialisation* and a *shared information system*. Tasks relating to care coordination, patient monitoring and maintaining patient records were transferred from general practitioners to case managers. Geriatric specialisation of general practitioners was a precondition for participation in the intervention. Postgraduate education programs for general practitioners in the Netherlands do not typically include geriatric care. Supplementary training was thus required to ensure that sufficient geriatric knowledge was available at intervention practices. Specifically, general practitioners gained insight into the associations between diseases and the daily functioning of frail elderly patients, and how to provide an integrated response to their needs by reshuffling tasks between primary, secondary and tertiary care. Case managers also received training in geriatric care, and completed courses on case management and the use of the evidence-based instruments. As well, a hospital geriatrician was available to intervention practices for consultation on complex cases. The shared information system allowed professionals to access and make adjustments to the care plan of a particular frail elderly patient.

### Data collection and instruments

#### Objective Burden

**Figure 2:**
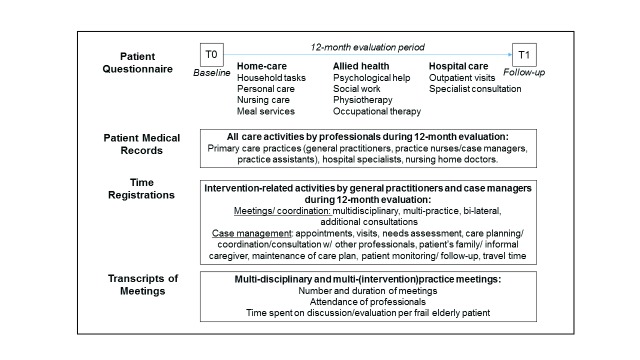
Objective burden data collection.

Data regarding the objective burden of professionals were collected from patient medical records, transcripts of multi-disciplinary meetings, time registrations of professionals and patient questionnaires. Written informed consent was obtained from all participating frail elderly patients. A total of 1845 frail elderly patients were approached, of which around 80% responded. Whilst 33% (n = 464) was subsequently identified as frail, a loss to follow-up of 19% between T0 and T1 resulted in a study population of 377 frail elderly patients, of whom the medical records were subsequently analysed (see [33] for further details). The medical records were extracted on location from the information systems of the 8 participating primary care practices. These data involved all care activities related to the delivery of care to each frail elderly patient over a 12-month period by general practice professionals (general practitioners, practice nurses, practice assistants and case managers), hospital specialists and nursing home doctors (Figure 2). Additionally, transcripts of multi-disciplinary meetings were used to determine which professionals had attended and the amount of time spent per frail elderly patient. General practitioners and case managers of intervention practices provided time registrations of intervention-specific activities, i.e., multi-disciplinary meetings, bimonthly joint meetings with all intervention practices, additional meetings with other primary care providers and hospital specialists, and bi-lateral meetings between general practitioners and case managers. Case managers also provided time registrations of activities relating to case management: making appointments/visiting patients, needs assessment, formulation of care plan, patient monitoring, follow-up visits/calls, care planning/coordination and travel time. The standardized registration forms were faxed to the researchers on a monthly basis. Finally, participating frail elderly patients were mailed a questionnaire at baseline and 12 months later regarding their use of home-care (domestic helpers, home-care nurses), allied health services (occupational-/ physiotherapist, social worker, psychologist) and hospital care services (Additional file 1).

Objective burden was differentiated into patient-related (visits, consultations) and non-patient related activities (meetings, prescriptions, referrals, administrative tasks) and linked to standardised time-units (Box 1). For primary care practice professionals, these time-units were based on the consensus among the general practitioners. For hospital specialists, time-units were obtained from the *Dutch Manual for Economic Evaluations* [34]. Time-units for patient-related activities of allied health professionals were obtained from professional associations and insurance companies. However, information regarding their non-patient related activities was unavailable.

**Box 1: Standardized time units in minutes per patient.**

**Table:** 

**Primary care practice professionals**	*Patient-related*		*Non-patient related*	
	Consultation by phone	5	Mail processing	0.5
	Consultation	10	Repeated prescription	3
	Double consultation	25	Consultation with general practitioner	5
	Visit	10	Consultation other practice	5
	Double visit	25	Consultation nursing home	5
	Visit (+30 min.)	35	Consultation specialist	5
	Other activities	0.5	Other consultations	0.5
**Hospital specialists**	*Patient-related*		*Non-patient related*	
Academic hospital	Outpatient consultation	15	Administration	5
General hospital	Outpatient consultation	10	Administration	3
**Allied health professionals**	*Patient-related*		Non-patient related^*^	
	Duration of session	30	–	
	Duration of session	60	–	

*Not available. Other activities = injections, glucose measurement, urine checks; other consultations = patient’s family, other professionals.

#### Job satisfaction

Data regarding the job satisfaction of professionals were collected with questionnaires that were distributed 18 months after implementation of the intervention (i.e., after all eligible elderly patients had been included). For privacy reasons, home-care organisations distributed the questionnaires to their own employees. Allocation of home-care professionals to the control or experimental group was based on their responses to additional items in their questionnaires regarding the location(s) at which they were (most) active as care providers. Employees of primary care practices were mailed a questionnaire and were allocated based on their affiliation to either a control or experimental group practice. As allied health and hospital professionals operate regionally, they could not be allocated to a group and were therefore excluded from the questionnaire study. The questionnaire was pilot-tested by a panel of 5 professionals (1 general practitioner, 3 registered nurses and 1 domestic helper). Based on their feedback, a case description of a frail elderly patient was included (Additional File 2). The case helped determine whether respondents were actually involved in the care for frail elderly patients. Respondents were asked to indicate whether they recognized the case in their daily work; if not, they did not have to fill out the questionnaire.

Job satisfaction was measured with the ‘Job Satisfaction Scale’, which has been validated in the healthcare setting reaching a high reliability (*α* = 0.86) [35]. Its 10 items address a range of intrinsic and extrinsic dimensions of job satisfaction and consist of a 7-point Likert scale from 1 (extremely dissatisfied) to 7 (extremely satisfied). Items regarding background variables included: age, gender, number of working hours per week, current professional role and number of years of experience in this role [35,36]. The questionnaire was designed according to the ‘post-then-pre’ principle, in which baseline and follow-up measurements are performed simultaneously [37].

### Analysis

In regard to objective burden, the time investments of all professionals were aggregated for each patient, and described using means, standard deviations and percentages. Within- and between-groups analyses were performed using paired or independent t-tests, McNemar’s test, Wilcoxon signed ranked test, Chi Square tests and Mann Whitney U-tests. Linear regression analyses were used to examine the extent and direction of potential associations between time investments, frailty and the intervention. The internal consistency of the job satisfaction scale was checked using Cronbach’s alpha, and missing values were imputed using the Expectation Maximization Method. All variables were described using means, standard deviations and percentages. The variable ‘current professional role’ was transformed into the dichotomous variable ‘employed by primary care practice’ to account for the central role of primary care practices in the development and implementation of the intervention. The new variable was included as a predictor in subsequent linear regression analyses. Regression analyses involved baseline scores (model 1), control variables (model 2) and the intervention (model 3). All models and effects were considered significant if *p* < 0.05.

## Results

### Professional questionnaire

Six hundred and twenty-six questionnaires were sent, of which 196 were returned. A total of 16 respondents were excluded due to their uninvolvement in the care to frail elderly patients (n = 10) or because they could not be allocated to the control or experimental group (n = 6). This amounted to 180 respondents and a response rate of 29%. The majority of respondents were female and employed by a home-care organisation, most of which working as domestic helpers (Table 1). The average age of respondents was 44 years. They worked around 21 hours per week and had been in their current positions for approximately 9 years. Whereas the experimental and control group were equal in terms of gender, age, years in current position and hours per week, the percentage of practice nurses/case managers and general practitioners was significantly higher in the experimental group.

**Table 1:** Questionnaire response and description of study population.

Response (*n/n*)	Professionals (*n* = 180)	Control group (*n* = 120)	Experimental group (*n* = 60)

Primary care (*28/48*)	General practitioner*	3	7
	Practice nurse/case manager*	3	5
	Practice assistant	5	5

Home-care (*152/578*)	Domestic helper	85	36
	Registered nurse	24	7

Control variables	Women (%)	97%	90%
	Men (%)	3%	10%
	Age (*M, SD*)	44.6 (12.7)	43.7 (11.6)
	Years in current position (*M, SD*)	9.1 (8.3)	8.4 (7.6)
	Hours per week (*M, SD*)	20.8 (9.6)	22.3 (11.5)

* p < 0.05; M = mean; SD = standard deviation.

### Job satisfaction

The internal consistency of the Job Satisfaction Scale was sufficient with values *α* = 0.82 (T0) and *α* = 0.75 (T1). Regression analyses showed that the intervention did not significantly affect job satisfaction (Table 2). Several control variables showed effects on separate dimensions of job satisfaction. Most notably, the number of work years negatively impacted the professionals’ satisfaction with the responsibilities (*p* = 0.004), physical conditions (*p* = 0.013), opportunities to use one’s own skills (*p* = 0.013), general work situation (*p* = 0.002), freedom of methods (*p* = 0.007) and number of work hours (*p* = 0.002). Age reduced professional satisfaction with colleagues (*p* = 0.001) but increased satisfaction with physical conditions (*p* = 0.005), the possibility to use personal skills (*p* = 0.037) and the number of work hours (*p* = 0.016). Professionals employed by practices were more satisfied with the responsibilities (*p* = 0.044) and variation in their work (*p* = 0.020). Baseline scores were a predictor for all dimensions of job satisfaction (*p* < 0.001).

**Table 2:** Linear regression analyses with job satisfaction scores (T1) as dependent variable, and baseline scores (T0), control variables and the intervention as independent variables.

		Predictors Baseline			Gender			Age			No. hours			No. years			Practice			Intervention		
								
Dimensions (1–7)	Adj.*R^2^*	B	SE	*β*	B	SE	*β*	B	SE	*β*	B	SE	*β*	B	SE	*β*	B	SE	*β*	B	SE	*β*

Responsibility	.15	0.32	0.06	**.36**	0.01	0.31	.00	0.01	0.01	.07	0.00	0.01	.01	–0.03	0.01	**–.24**	0.37	0.18	**.16**	–0.07	0.13	–.04
Variation	.27	0.53	0.07	**.51**	0.36	0.31	.09	0.01	0.01	.08	0.01	0.01	.05	–0.01	0.01	–.09	0.44	0.19	**.17**	–0.07	0.13	–.04
Colleagues	.40	0.43	0.05	**.52**	0.09	0.30	.02	–0.02	0.01	**–.23**	0.01	0.01	.10	–0.01	0.01	–.09	0.30	0.18	.11	–0.02	0.13	–.01
Physical conditions	.59	0.69	0.05	**.73**	–0.21	0.28	–.05	0.02	0.01	**.16**	–0.01	0.01	–.06	–0.02	0.01	–.11	0.25	0.17	.09	0.21	0.12	.09
Skills	.54	0.77	0.06	**.73**	–0.19	0.31	–.05	0.01	0.01	**.13**	0.00	0.01	.01	–0.02	0.01	**–.16**	0.29	0.19	.09	0.10	0.13	.04
General situation	.23	0.45	0.07	**.44**	–0.01	0.01	–.02	0.01	0.01	.08	0.00	0.01	.03	–0.03	0.01	**–.25**	0.25	0.20	.10	0.17	0.14	.09
Freedom	.39	0.46	0.04	**.63**	–0.04	0.19	–.02	0.01	0.00	.14	0.00	0.01	.05	–0.02	0.01	**–.20**	–0.02	0.12	–.01	0.08	0.08	.06
Appreciation	.61	0.76	0.05	**.77**	0.38	0.27	.08	0.01	0.01	.06	0.01	0.01	.07	–0.00	0.01	–.03	0.14	0.16	–.05	–0.07	0.12	–.03
Remuneration	.82	0.92	0.03	**.92**	0.08	0.24	.01	–0.00	0.01	–.03	–0.00	0.01	–.02	0.00	0.01	.02	–0.06	0.15	–.02	–0.04	0.10	–.01
Hours	.75	0.84	0.04	**.86**	0.10	0.21	.02	0.10	0.00	**.11**	–0.00	0.01	–.02	–0.02	0.01	**–.14**	0.21	0.13	.07	–0.05	0.09	–.02
Total satisfaction	.37	0.53	0.05	**.61**	–1.26	1.80	–.05	0.02	0.03	.04	–0.01	0.04	–.02	–0.07	0.05	–.10	–1.06	1.07	–.07	–1.02	0.76	–.08

Adj.R^2^ = Adjusted Explained Variance; B = Unstandardized Coefficient; SE = standard error; β = Standardized Coefficient (Bold = p < 0.05). All reported regression models were significant (p < 0.001).

### Objective burden

There was no significant difference between the control and experimental group in the mean total time investment and patient-related time investment of all professionals combined (Table 3). The mean non-patient related time investment of all professionals was, however, significantly higher in the experimental group than in the control group (t(375) = –21.947, *p* = 0.000). The mean of total time investments (t(375) = –3.149, *p* = 0.002) and non-patient related time investments (t(375) = –9.464, *p* = 0.000) of professionals of primary care practices (excluding case managers) were significantly higher in the experimental group, whereas no differences in patient-related time investment were observed. Similarly, the mean total time investment (t(375) = –6.231, *p* = 0.000) and mean non-patient related time investment (t(350) = –18.477, *p* = 0.000) of general practitioners was significantly higher in the experimental group, but there was no significant difference in their patient-related time investment. Practice assistants in the control group had significantly higher mean total (t(226.450) = 4.371, *p* = 0.000), patient-related (t(229.267) = 3.492, *p* = 0.001) and non-patient related (t(227.954) = 4.184, *p* = 0.000) time investments than practice assistants in the experimental group. Conversely, practice nurses in the experimental group had significantly higher mean total (t(318.930) = –3.573, *p* = 0.000), patient-related (t(305.667) = –3.327, *p* = 0.001) and non-patient related (t(349.917) = –3.437, *p* = 0.001) time investments than practice nurses in the control group. Finally, there were no significant differences between the experimental and control group in the time investments of hospital specialists, home-care professionals and allied health professionals.

**Table 3:** Mean and Standard Deviation of professionals’ time investments in minutes per frail elderly patient over the 12-month evaluation period for the control and experimental group.

	Control group (*n* = 193 patients)						Experimental group (*n* = 184 patients)					
		
	Total		Patient-related		Non-patient related		Total		Patient-related		Non-patient related	
	
Type of professional	*M*	*SD*	*M*	*SD*	*M*	*SD*	*M*	*SD*	*M*	*SD*	*M*	*SD*

All professionals	11547	15733	11517	15726	**30**	41	12926	14439	12576	14433	**350**	194
Home-care	10989	15490	**–**	**–**	**–**	**–**	11833	14265	**–**	**–**		**–**
Allied health	369	907	**–**	**–**	**–**	**–**	352	1054	**–**	**–**		**–**
Hospital specialist	23	24	18	19	5	6	23	29	18	22	5	7
Primary care practice*	**165**	159	140	130	**25**	40	**212**	129	146	103	**66**	45
Case manager	**–**	**–**	**–**	**–**	**–**	**–**	230	281	115	140	115	151
General practitioner	**86**	76	83	73	**3**	7	**136**	81	86	66	**51**	34
Practice assistant	**52**	86	**32**	65	**19**	35	**24**	25	**15**	20	**9**	10
Practice nurse	**27**	47	**25**	40	**2**	9	**49**	69	**43**	63	**6**	12

*Excluding case managers. M = Mean; SD = standard deviation. Bold = p < 0.05.

Regression analyses were performed to examine the contribution of the intervention and patients’ frailty to each type of time investment (Table 4). Frailty showed significant relationships with total, (*p* = 0.000), patient-related (*p* = 0.000) and non-patient related (*p* = 0.008) time investments of all professionals combined, whereas the intervention only showed significance on non-patient related time investments (*p* = 0.008). Frailty was significantly associated with total (*p* = 0.009) and non-patient time investments (*p* = 0.018) of primary care practice professionals, and the intervention with their total (*p* = 0.003) and non-patient related (*p* = 0.000) time investments. For general practitioners, frailty was related to total (*p* = 0.000), patient-related (*p* = 0.001) and non-patient related time investment (*p* = 0.031). The intervention showed significance on total (*p* = 0.000) and non-patient related time investment (*p* = 0.000) of general practitioners. No relationships were found between frailty and the time investments of both practice assistants and nurses, whilst the intervention showed significance in all 3 categories of time investment for both types of professionals (*p* ≤ 0.001). These effects were negative for practice assistants and positive for practice nurses. Frailty was significantly associated with home-care professionals’ total time investments (*p* = 0.000).

**Table 4:** Linear regression analyses with total, patient-, and non-patient related time investments as dependent variables, and frailty and the intervention as independent variables.

		Time-investments											
		
		Total				Patient-related				Non-patient related			
				
Type of professionals	Predictor	*Adj. R^2^*	B	SE	*β*	*Adj. R^2^*	B	SE	*β*	*Adj. R^2^*	B	SE	*β*

All professionals	Frailty	0.12	2802	393	**.346**	0.12	2792	393	**.345**	0.02	10	4	.**089**
	Intervention	0.12	665	1466	.022	0.12	348	1467	.012	0.58	317	14	**.751**
Primary care practice	Frailty	0.02	10	4	**.134**	n.s.	–	–	–	0.02	3	1	**.110**
	Intervention	0.04	44	15	**.151**	n.s.	–	–	–	0.20	41	4	**.432**
General practitioner	Frailty	0.04	8	2	**.176**	0.03	6	2	**.169**	0.01	2	1	**.080**
	Intervention	0.12	49	8	**.294**	0.02	1	7	.011	0.49	47	3	**.699**
Practice assistant	Frailty	0.00	2	2	.060	0.00	1	1	.047	0.00	1	1	.060
	Intervention	0.05	–29	7	**–.220**	0.03	–18	5	**–.177**	0.04	–11	3	**–.211**
Practice nurse	Frailty	0.00	0	2	.010	0.00	0	1	–.001	0.00	0	0	.059
	Intervention	0.03	22	6	**.182**	0.02	18	5	**.171**	0.03	4	1	**.172**
Home-care	Frailty	0.11	2720	389	**.341**	–	–	–	–	–	–	–	–
	Intervention	0.11	152	1449	.005	–	–	–	–	–	–	–	–
Hospital	Frailty	n.s.	–	–	–	n.s.	–	–	–	n.s.	–	–	–
	Intervention	n.s.	–	–	–	n.s.	–	–	–	n.s.	–	–	–
Allied health	Frailty	n.s.	–	–	–	–	–	–	–	–	–	–	–
	Intervention	n.s.	–	–	–	–	–	–	–	–	–	–	–

Adj.R^2^ = Adjusted Explained Variance; B = Unstandardized Coefficient; SE = standard error; β = Standardized Coefficient (Bold = p < 0.05); n.s.= regression model not significant. *Note*: Values of B and SE are rounded up.

Overall, frailty explained little variance, whereas contribution of the intervention was considerable in non-patient related time investments of all professionals, general practitioners, and primary care practice professionals (from 1–2% of variance explained to 58%, 49% and 20%, respectively).

## Discussion

The aim of this study was to determine the impact of integrated working on professionals’ objective burden and job satisfaction in the context of an intervention targeting frail elderly patients in the community. To our knowledge, this study is the first to use data from formal administrative systems to do so. The results demonstrate that professionals delivering care according to the Walcheren Integrated Care Model spent significantly more time on non-patient related activities than professionals delivering usual care, whereas no differences were found in time spent on patient-related activities. As well, professionals’ job satisfaction was not affected by the intervention.

These findings confirm a major concern among scholars and professionals regarding the impacts of integrated working. It is widely believed that patient-centred care requires additional coordination activities on top of regular practice routines [8,9,10,16,17,18]. Leutz [10] noted that ‘integration costs before it pays’, and many in his wake have noted that the up-front investments of integrated care are unavoidable, whereas the future pay-off is uncertain [2,9,10,11,38]. The transition towards integrated working is also believed to be a long-term process from which no short-term efficiency gains can realistically be expected [26]. The present study confirms that this is indeed the case, at least in the first 12 months of integrated working. However, given enough time, integrated working may prove beneficial, as it increasingly becomes a practice routine [11,12,13,38].

In the present study, professionals from practices that delivered integrated care spent more time on non-patient related activities than professionals from practices that delivered usual care. Whilst this finding may not be surprising considering the additional intervention- and case management-related activities, it does raise questions regarding the long-term sustainability of integrated care. General practitioners already face considerable workloads, to which the responsibility of integrating care for specific patient subgroup only adds [10]. As well, intervention-related activities may supplant existing workloads of general practitioners and their staff. Professionals may feel compelled to work overtime at increasing personal cost just to maintain progress in the intervention [27]. Consequently, integrated care may cause staff burnout and retention problems, affecting its sustainability in the long run [7,11,27,29]. Several authors have therefore called for better support of practices developing integrated care to offset the detrimental impacts on staff, for instance by providing additional financial and human resources [13,18,22,38,39,40,41,42].

Intervention practice assistants had lower time investments than their counterparts in the control group, whereas the opposite pattern was observed in practice nurses. These small but significant effects suggest a transfer of certain tasks from assistants to practice nurses due to integrated working, perhaps signifying a process of task-redistribution that may persist well beyond the 12-month evaluation period.

No significant difference in time spent by home-care professionals was observed between the experimental and control group, which likely reflects their lack of direct involvement in the intervention. Relatively little is known about the extent to which home-care professionals should be involved in integrated care for the frail elderly. Service users often report strong bonds with their home-care worker [43], which, in itself, may be ample justification for their involvement. However, home-care professionals are typically less qualified than primary care practice staff, which may hamper collaboration and the development of a common understanding of care for frail elderly patients [44].

Job satisfaction remained unchanged by the intervention. As the majority of respondents were home-care professionals, integrated working may have had little impact outside the practices at which it was implemented. Job satisfaction is largely insensitive to changes in the organisation of care delivery, particularly if the work itself remains much the same [36]. The lack of effect on job satisfaction can also be interpreted as a positive result when considered in conjunction with the observed increase in objective burden. Professionals are typically sceptical towards new ways of working due to the additional time investments implied [23]. Non-patient related time investments in particular have been linked to low job satisfaction [14]. However, the use of different populations and data collection methods in the present study allows no definitive conclusions regarding an association between objective burden and job satisfaction in the integrated care context.

### Strengths and limitations

The main strengths of this study are the collection of objective data from formal systems in combination with information from other sources, and the use of a validated measure of job satisfaction. The main limitation was the relatively low response to the professional questionnaire. One explanation is that only a subset of potential respondents was actually involved in care for frail elderly patients. As well, recall over an 18-month period may not be entirely accurate. The ‘pre-then-post’ design can lead to socially desirable responses, although this seems to be outweighed by its advantages, i.e., a minimum time investment for respondents and reduced risk of response shift bias [37]. Self-reporting methods such as time registration forms are prone to inaccuracy, but are the only means of documenting intervention-related activities that are not documented elsewhere. Moreover, these forms provided only a fraction of objective burden data, the bulk of which was derived from patient medical records.

### Recommendations for research and practice

Future research should focus on long-term impacts of integrated working on the objective burden and job satisfaction of professionals. Of particular interest is whether the initial time demands diminish over time and if the benefits for professionals, most notably job satisfaction, become apparent. Interactions between job satisfaction and objective burden could be examined by collecting data on both outcomes from a single population of professionals. Ideally, these data are collected over multiple years to determine how integrated working affects the process of task distribution over time. A longitudinal approach allows in-depth analyses of the contribution of separate integrated care components to objective burden. Furthermore, future research should address the role and involvement of home-care professionals in integrated care models for frail elderly patients.

This study begs the question whether the general practitioner should invariably be the ‘chief integrator’ and single-entry point. As the gatekeepers to the health system, general practitioners seem optimally positioned to lead integration efforts in the Netherlands. The central role of general practitioner is, however, not universal, and other professionals may be better equipped to drive integrated care efforts in other countries. Still, regardless of the setting, integrated care for the frail elderly is a complex undertaking that involves patients with complex care demands. It is therefore recommendable that the final responsibility of these initiatives rests with a medical doctor. Finally, integrated care is unlikely to produce short-term efficiency gains whilst almost certainly placing additional burden on professionals. Integrated care planning and practice should therefore be based on realistic expectations regarding its costs and should explicitly address staff well being and support.

## Supplementary Files

The supplementary files for this article can be found as follows:

- **Supplementary File 1: Additional File 1. **ijic-16-3-2014-s1.pdf
- **Supplementary File 2: Additional File 2. **ijic-16-3-2014-s2.pdf
