# Supplementary material for: Delivering Integrated Care to the Frail Elderly: The Impact on Professionals’ Objective Burden and Job Satisfaction
Source: Int J Integr Care. 2016 Aug 17;16(3):7. doi: 10.5334/ijic.2014 (PMC5374987; doi:10.5334/ijic.2014)
Supplement: Supplementary file 1 [file ijic-16-3-2014-s1.pdf]

QUESTIONNAIRE

Care receiver

Your experiences as a care receiver are valuable

You receive care. This list contains questions about what this care means for you as a care receiver. Your answers will be used in research to improve care for the elderly. The more information there is about care for the elderly, the more this can be taken into consideration. For example when changing the facilities, regulations or legislation for care and wellbeing. Therefore, your experiences are also valuable for other elderly people. Your answers will be processed anonymously and your data will not be distributed further.
If you complete this questionnaire, this will not have any effect on the care that you receive.

If you have any further questions, please call us on ...

Instructions for this questionnaire

- Completing this questionnaire will take approximately half an hour.
- Read each question through completely before selecting an answer.
- Then tick the answer that best fits your situation.
- Tick only one answer for each question.
- If you are allowed to tick more than one answer, then this will be mentioned for the relevant question.
- If you find it difficult to understand or answer the questions, ask your partner, a family member or a friend to help you.
- Some questions may appear to be ‘repeated’, but please answer all the questions. They are intended to view your situation again from a different angle.
- When you are done, please check that you have not forgotten any questions.

Date of birth, gender and postal code

Please fill in your details below:

1 Date of birth: - -

2 Gender:

- ☐ Male
- ☐ Female

3 Postal code: | | | | | *Only fill in the four figures here*

Personal information

4 In which country were you born?

- ☐ The Netherlands
- ☐ Another country: ______________

5 In which country was your father born?

- ☐ The Netherlands
- ☐ Another country: ______________

6 In which country was your mother born?

- ☐ The Netherlands
- ☐ Another country: ______________

7 What is the highest level of education that you have completed?

- ☐ Fewer than 6 years of primary school
- ☐ 6 years of primary school, lom school, mlk school (special education)
- ☐ More than primary school / primary school without further completed education
- ☐ Vocational school
- ☐ Mulo / mms / mavo / secondary professional education
- ☐ Hbs / gymnasium / atheneum (university entrance level)
- ☐ University / tertiary education

8 What is your marital status?

- ☐ Married
- ☐ Divorced
- ☐ Widow / widower / partner deceased
- ☐ Unmarried

- ☐ Long-term cohabitation, unmarried

Living situation

9 What is your living situation?

- ☐ Independent, alone
- ☐ Independent, with others (partner, children, etc.)
- ☐ Care home / residential care centre since ________ - ____ - ____
- ☐ Nursing home since ________ - ____ - ____

Health and illnesses

The following questions are about your health. Please tick the box of the answer that best fits your situation.

10 How is your health in general?

- ☐ Excellent
- ☐ Very good
- ☐ Good
- ☐ Reasonable
- ☐ Poor

11 How is your health in general, in comparison to one year ago?

- ☐ Much better
- ☐ Slightly better
- ☐ About the same
- ☐ Slightly worse
- ☐ Much worse

The following questions are about how you are feeling today. Tick the box next to the sentence that best describes your health right now.

12 Walking

- ☐ I have no problems with walking
- ☐ I have some problems with walking
- ☐ I am bedridden

13 Self care

- ☐ I have no problems washing or dressing myself
- ☐ I have some problems washing or dressing myself
- ☐ I am unable to wash or dress myself

14 Daily activities (for example work, education, household, family and leisure activities)

- ☐ I have no problems with my daily activities
- ☐ I have some problems with my daily activities
- ☐ I am unable to perform my daily activities

15 Pain / symptoms

- ☐ I have no pain or other symptoms
- ☐ I have moderate pain or other symptoms
- ☐ I have very severe pain or other symptoms

16 Mood

- ☐ I am not anxious or despondent
- ☐ I am moderately anxious or despondent
- ☐ I am very anxious or despondent

17 Brain functions such as memory, attention and thinking

- ☐ I have no problems with my memory, attention and thinking
- ☐ I have some problems with my memory, attention and thinking
- ☐ I have severe problems with my memory, attention and thinking

The following questions are about the illnesses and conditions that you have or have had.

18 Place a tick next to the illnesses and conditions that you have at the moment or have had in the past 12 months. You can select more than one answer.

- ☐ Diabetes
- ☐ Stroke, cerebral haemorrhage (bleed in the brain), cerebral infarction (blocked blood vessel in the brain) or TIA
- ☐ Heart failure
- ☐ A type of cancer (malignant condition)
- ☐ Asthma, chronic bronchitis, lung emphysema or COPD
- ☐ Involuntary loss of urine (incontinence)
- ☐ Wearing of the joints (arthrosis, degenerative arthritis) of hips or knees
- ☐ Loss of bone tissue (osteoporosis)
- ☐ Broken hip
- ☐ Broken bones other than a broken hip
- ☐ Dizziness with falling
- ☐ Prostate symptoms caused by benign prostate enlargement
- ☐ Depression
- ☐ Anxiety / panic disorder
- ☐ Dementia
- ☐ Hearing problems
- ☐ Problems with vision

Tasks and activities of daily life

The following questions are about how you function in daily life. Select your answer according to the situation as it is now. Please tick the box of the answer that best fits your situation.

19 Do you need help with bathing or showering?

☐ no

☐ yes

20 Do you need help with getting dressed?

☐ no

☐ yes

21 Do you need help with combing your hair or with shaving?

☐ no

☐ yes

22 Do you need help with going to the toilet?

☐ no

☐ yes

23 Do you use incontinence products?

☐ no

☐ yes

24 Do you need help to get up out of a chair?

☐ no

☐ yes

25 Do you need help with walking?

☐ no

☐ yes

26 Do you need help with eating?

☐ no

☐ yes

27 Do you need help with using the telephone?

☐ no

☐ yes

28 Do you need help with travelling?

☐ no

☐ yes

29 Do you need help with grocery shopping?

☐ no

☐ yes

30 Do you need help with preparing a meal?

☐ no

☐ yes

31 Do you need help with household tasks?

☐ no

☐ yes

32 Do you need help with taking your medicines?

- ☐ no
- ☐ yes

33 Do you need help in dealing with finances?

- ☐ no
- ☐ yes

How you are feeling

The following questions are about how you have been feeling the past month. Please tick the box of the answer that best fits your situation.

34 How often in the past month have you been very nervous?

- ☐ Always
- ☐ Very often
- ☐ Quite often
- ☐ Sometimes
- ☐ Almost never
- ☐ Never

35 How often in the past month have you felt calm and tranquil?

- ☐ Always
- ☐ Very often
- ☐ Quite often
- ☐ Sometimes
- ☐ Almost never
- ☐ Never

36 How often in the past month have you felt despondent and sombre?

- ☐ Always
- ☐ Very often
- ☐ Quite often
- ☐ Sometimes
- ☐ Almost never
- ☐ Never

37 How often in the past month have you felt happy?

- ☐ Always
- ☐ Very often
- ☐ Quite often
- ☐ Sometimes
- ☐ Almost never
- ☐ Never

38 How often in the past month have you felt so sombre that nothing could cheer you up?

- ☐ Always
- ☐ Very often
- ☐ Quite often
- ☐ Sometimes
- ☐ Almost never
- ☐ Never

Social activities

39 How often ***in the past 4 weeks*** have your physical health or emotional problems hampered your social activities (such as visits to friends or close family members)?

☐ Continuously

☐ Mostly

☐ Sometimes

☐ Rarely

☐ Never

Quality of life

The following questions are about your ‘quality of life’. This refers to what you think about your life. For example, whether you are satisfied with your life, whether you have enjoyment in your life and whether your life gives you satisfaction. Please tick the box of the answer that best fits your situation.

40 How is your quality of life in general?

- ☐ Excellent
- ☐ Very good
- ☐ Good
- ☐ Reasonable
- ☐ Poor

41 Which report mark would you give your life at this moment?

Report mark: ☐ *Enter a figure between 0 and 10 here*

42 How is your quality of life in general, in comparison to one year ago?

- ☐ Much better
- ☐ Slightly better
- ☐ About the same
- ☐ Slightly worse
- ☐ Much worse

Your care use

43 Have you been admitted to a hospital in the past 12 months?

- ☐ No
- ☐ Yes, namely ☐ days in total

If yes, in which hospitals?

Admission 1

Hospital: ________________

City: ________________

Admission 2

Hospital: ________________

City: ________________

Admission 3

Hospital: ________________

City: ________________

Admission 4

Hospital: ________________

City: ________________

Admission

5

Hospital:

City:

44 Have you visited an out of hours GP service or had a visit from a general practitioner in the evening, night or on the weekend for yourself in the past 12 months?

☐ No

☐ Yes, namely ☐ times in total

45 Do you receive home care? For example a community nurse, family care or home help.

☐ No

☐ Yes, namely ☐ hours per week

46 Have you been admitted to a care home **temporarily** in the past 12 months? For example because you were unable to go home immediately after a hospital admission.

☐ No

☐ Yes, namely ☐ weeks in total

47 Have you been admitted to a nursing home **temporarily** in the past 12 months? For example because you were unable to go home immediately after a hospital admission.

☐ No

☐ Yes, namely ☐ weeks in total

48 Do you go to a day care centre?

☐ No

☐ Yes, namely ☐ days per week

49 Do you go for day treatment?

- ☐ No
- ☐ Yes, namely ☐ days per week

Conclusion

50 Has somebody helped you to complete this questionnaire?

- ☐ No, I completed the list alone.
- ☐ Yes, somebody helped me to complete the list.

51 If yes, what did the help consist of?

- ☐ Someone else recorded the answers, but I selected the answers myself.
- ☐ I selected and recorded the answers together with someone else.
- ☐ Someone else selected and recorded the answers for me.

If you have any comments, please write them down in the space below:

This is the end of the questionnaire. Thank you very much for completing the questionnaire!!!
