# Supplementary material for: Delivering Integrated Care to the Frail Elderly: The Impact on Professionals’ Objective Burden and Job Satisfaction
Source: Int J Integr Care. 2016 Aug 17;16(3):7. doi: 10.5334/ijic.2014 (PMC5374987; doi:10.5334/ijic.2014)
Supplement: Supplementary file 2 [file ijic-16-3-2014-s2.pdf]

Additional File 2. Case description of a frail elderly patient

This case describes the care situation of a 'typical' frail elderly patient. Please read the description before filling out the questionnaire. You may not encounter this type of patients (regularly) in your daily work as a care professional. If so, please check the box below: you do not have to fill out the questionnaire.

☐ *I do not encounter this type of patients in my daily work as a care professional*

Mrs. A. is 83 years of age and widowed. She never really recovered from the loss of her husband two years ago. Mrs. A.'s 4 adult children each live in different regions, and only the oldest daughter visits her weekly. The daughter recently noticed that Mrs. A. seemed increasingly disoriented and forgetful. After consultation with the GP, Mrs. A was referred to the hospital for neurological and neuropsychological assessment. Mrs. A. was diagnosed with Alzheimer's disease shortly thereafter. Meanwhile, Mrs. A. seemed increasingly unable to adequately manage her (diabetic and heart failure) medication regimen. The GP therefore asked the practice's diabetic nurse specialist to visit Mrs. A. once a month. Mrs. A. received daily household assistance and 'Meals on Wheels' from a home-care organization.
One day, Mrs. A. fractured her hip by tripping over the living room rug. Recovery was slow, and Mrs A. remained largely immobile after returning home. During this period, home-care professionals noticed a worsening of Mrs. A. depressive symptoms. Concerned for the safety and well being of her mother, the oldest daughter applied for a nursing home to which Mrs. A. was admitted within months.
